# Ultrasound Flow Imaging Study on Rat Brain with Ultrasound and Light Stimulations

**DOI:** 10.3390/bioengineering11020174

**Published:** 2024-02-10

**Authors:** Junhang Zhang, Chen Gong, Zihan Yang, Fan Wei, Xin Sun, Jie Ji, Yushun Zeng, Chi-feng Chang, Xunan Liu, Deepthi S. Rajendran Nair, Biju B. Thomas, Qifa Zhou

**Affiliations:** 1 Department of Biomedical Engineering, University of Southern California, Los Angeles, CA 90089, USA; 2 Department of Ophthalmology, USC Roski Eye Institute, Keck School of Medicine of the University of Southern California, Los Angeles, CA 90033, USA; 3 Caruso Department of Otolaryngology-Head & Neck Surgery, University of Southern California, Los Angeles, CA 90033, USA

**Keywords:** ultrasound stimulation, retinal stimulation, blood flow imaging

## Abstract

Functional ultrasound (fUS) flow imaging provides a non-invasive method for the in vivo study of cerebral blood flow and neural activity. This study used functional flow imaging to investigate rat brain’s response to ultrasound and colored-light stimuli. Male Long-Evan rats were exposed to direct full-field strobe flashes light and ultrasound stimulation to their retinas, while brain activity was measured using high-frequency ultrasound imaging. Our study found that light stimuli, particularly blue light, elicited strong responses in the visual cortex and lateral geniculate nucleus (LGN), as evidenced by changes in cerebral blood volume (CBV). In contrast, ultrasound stimulation elicited responses undetectable with fUS flow imaging, although these were observable when directly measuring the brain’s electrical signals. These findings suggest that fUS flow imaging can effectively differentiate neural responses to visual stimuli, with potential applications in understanding visual processing and developing new diagnostic tools.

## 1. Introduction

The human visual system is a sophisticated yet marvelous network that processes information through multiple layers. First perceived by photoreceptors in the retina, optical signals are transformed into electrical signals that are transferred to the central nervous system via the optic nerve. An array of subcortical structures, including the lateral geniculate nucleus (LGN), then integrate this information before sending it to the Visual Cortex (VC) for more advanced processing that eventually enables the perception of shapes, colors, and motion, and they ultimately form the coherent visual experience that is fundamental to interaction with our environment [1]. While extensive details about visual perception under healthy and diseased conditions have been revealed through histology and electrophysiology measurements such as electroretinography (ERG) [2], a complete understanding of the signal processing cascade remains challenging. Functional imaging methodologies with a higher spatial resolution are critically needed to further dissect and comprehend the visual pathway. 

High-resolution in vivo light stimulation and optical imaging on rodent retinas have been applied to study the complex mechanisms of the visual system and eye function. The single-cell fluorescence imaging of a stimulated mouse’s retina was achieved with a scanning laser microscope [3]. Further potentiating high-resolution retinal imaging, a recently published apparatus utilized a head-restrained preparation for awake mice, enabling high-quality imaging without anesthesia [4]. Functional intrinsic optical signal imaging or opto-retinography (ORG) is another high-resolution technique that can provide a quantitative evaluation of photoreceptor activities [5].

However, these studies are focused on the retinal regions. To better understand the elaborate visual system, we must go beyond the initial stages of signal capture and dissect the nuanced pathways through which these signals inform cerebral activity by relating retinal stimulation to brain imaging. Functional magnetic resonance imaging (fMRI) with Blood-Oxygen Level-Dependent (BOLD) imaging can perform a non-invasive 3D mapping of brain activity, providing a scope to study the complicated system-level hemodynamic and metabolic effects of retinal stimulation [6,7,8]. However, extending human MRI techniques to pre-clinical rodent disease models is challenging due to the smaller structures of the rodent brain. The cost for increasing the resolution would be the need for a strong magnetic field that usually comes with a cumbersome size that can limit experimental design. 

Functional ultrasound (fUS) imaging can potentially balance the need for resolution and the ease of implementation. It has emerged from ultrafast ultrasound imaging, which raised a more-than-400-time increase in image acquisition rate, up to 20,000 frames per second [9,10,11,12,13,14,15]. The temporal resolution of Power Doppler imaging is, thus, significantly improved, with the measurement of cerebral blood volume (CBV) possible in much smaller blood vessels [16]. Combined with the high spatial resolution of miniaturized high-frequency transducers, long-term hemodynamic changes in freely moving rodents can be coupled to specific stimulation signals [17,18,19]. Furthermore, ultrasound also serves as the stimulation signal to induce functional change. Being non-invasive, ultrasound has the advantage over electrical and optical stimulation in modulating neuronal activities throughout the visual pathway from the retina to the cortex [20,21]. 

Combining the benefits of ultrasound as both stimulation and imaging sources, in this study, we used fUS imaging to investigate the response of the visual system in rat brain to ultrasound and different colored-light stimuli in the retina. Activation maps of the visual cortex and LGN were generated by calculating the correlation coefficient (r) between the ultrasound power Doppler signals obtained through fUS and the temporal pattern of visual stimuli. Additionally, we measured the CBV response to different light stimulations within the visual system. 

## 2. Materials and Methods

### 2.1. Animal Preparation

This in vivo study was performed using male Long-Evan (LE) rats weighing 250 to 350 g. The University of Southern California Institutional Animal Care and Use Committee approved all the procedures involving the animals. These animals were maintained in pairs in each cage, having unrestricted access to food and water. The environment was controlled with a 12 h cycle of light and darkness at a temperature of 22 °C. Anesthesia was initiated with an intraperitoneal injection of a Ketamine/Xylazine mixture, followed by inhalation of sevoflurane delivered through a nose cone [22]. A heating pad was placed underneath the animal, ensuring that the body temperature was maintained at 37 °C throughout the experiment. The rats were secured in a stereotaxic apparatus during the surgical and imaging procedures. Then, a sagittal incision was made on the posterior region of the head to expose the scalp, followed by the creation of a rectangular craniotomy. Afterward, the bone was carefully separated from the dura mater to expose a part of the cortex that covers the visual cortex area that stretches up to 13 mm wide from Bregma −2 to −8 mm. All experimental procedures were conducted in a room with dim red lighting to prevent the bleaching of the photoreceptors. Before ultrasonic imaging, an ultrasound gel, which had been centrifuged to eliminate bubbles, was used to fill the gap between the brain surface and the transducer.

### 2.2. Transducer Design and Acoustic Field

**Figure 1:**
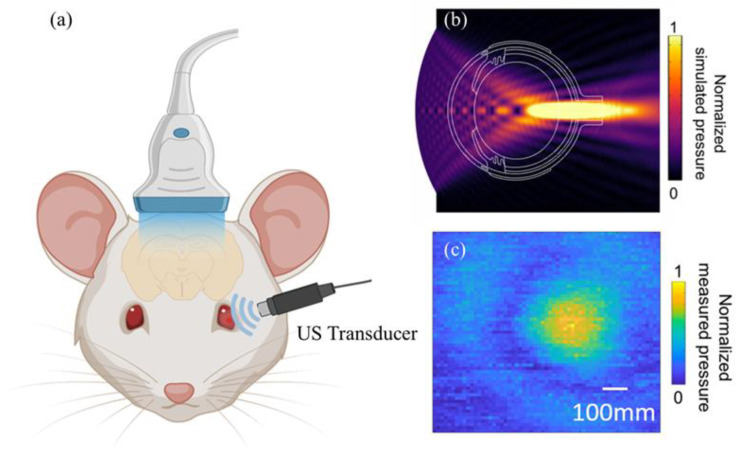
Schematic diagram of (**a**) ultrasound stimulation of the rat eye and fUS imaging of the visual areas of the rat brain, (**b**) COMSOL Multiphysics simulation of the transducer used for retinal stimulation, and (**c**) Hydrophone measurement of the ultrasound stimulation transducer.

A 3.5 MHz transducer with a focal depth of 8 mm was used for ultrasound retinal stimulation. The sound field distribution of the stimulation transducer was simulated with COMSOL Multiphysics software, version 6.1, shown in Figure 1b. The transverse profiles of the transducer were measured with a needle hydrophone (HGL-0400, ONDA, Sunnyvale, CA, USA). First, the transducer and hydrophone were mounted onto a three-axis motorized displacement stage. The motor drove the hydrophone to scan the XY plane. The hydrophone signal was amplified using a preamplifier and input to a computer acquisition card that sampled at 1800 MHz. The transducer was set up with an input of a 3.5 MHz sine wave in burst mode, a 30 V amplitude, and 20 cycles. MATLAB (MATLAB Release 2022, The MathWorks, Natick, MA, USA) was used to analyze the measurement results of the transducer, which are shown in Figure 1c. The focal point size of the transducer was 500 μm.

### 2.3. Electrophysiological Signal Acquisition

EEG signals were recorded from the head using a circular multi-electrode array (2 MΩ, tungsten-standard tip-with polyimide tube, MicroProbes, Gaithersburg, MD, USA) inserted into the visual cortex of the rats. A reference electrode was placed 3 mm posterior to the lambda, and a ground electrode was positioned on the scalp skin. The eye was coupled to a 3.5 MHz. transducer. Ultrasound stimulation was administered to the rats’ eyeballs at two output voltages, 30 Vpp and 90 Vpp, for a 50 ms duration with a 1 s interval. A function generator was used to synchronize the DAQ system and ultrasound transducer. The electrophysiological signals were captured using a probe (Plexon, Dallas, TX, USA, 20X, probe), subsequently amplified with a preamplifier (Plexon, Dallas, TX, USA, 50X, bandpass filter 133 Hz–10 kHz), and finally recorded by a PowerLab(ADInstruments, Sydney, Australia) data acquisition (DAQ) system (sampling rate 40 kHz, Powerlab 8/30 and LabChart, ADInstruments, Sydney, Australia). A function generator (AFG 3252C, Tektronix, Beaverton, OR, USA) was used to synchronize the DAQ system and ultrasound transducer.

### 2.4. Light and Ultrasound Stimulation Protocols

In the comparative experiment assessing the effects of light and ultrasound stimulations on the retina, imaging was performed on five male Long-Evan (LE) rats. To record light stimulation activities in the visual system, a full-field strobe flash using a Grass Photic stimulator (Grass Instrument Co., W. Warwick, RI, USA) was placed 8 cm away from the eye to deliver light stimulation. A Verasonics system(Vantage 256, Seattle, WA, USA) was synchronized with the Grass Photic stimulator using a trigger out function. The light stimulation protocol used in this experiment included two white light stimulation (no filters used) groups and three multi-color stimulation (filters used) groups. For the first white light stimulation group, the light stimuli were delivered to both eyes, while in the second group, only the left eye received the stimulation. For the multi-color stimulation groups, blue (wavelength of 460 nm), yellow (wavelength of 570 nm), or red (wavelength of 650 nm) filters were placed in front of the light source, allowing the rats to receive specific wavelengths of light stimulation in both eyes. 

During the ultrasound stimulation, the ultrasound probe was positioned on the surface of the eye with an ultrasound gel applied between the probe and the cornea to enhance the coupling of the ultrasound signal. The schematic diagram of the US stimulation on the rat retina is shown in Figure 1a. The parameters used for US stimulation were the same as those used for EEG recording but with a voltage of only 90 Vpp. Our ultrasound probe was designed to have a focal distance of 8 mm, which is similar to the size of rat eye [23], allowing for the ultrasound energy to precisely focus on the rats’ retinas. Both the light and ultrasound stimulation protocols were designed to activate vision-involved brain structures [24], including the visual cortex, superior colliculus, and lateral geniculate nucleus (LGN).

### 2.5. Ultrasound Flow Imaging Sequence

**Figure 2:**
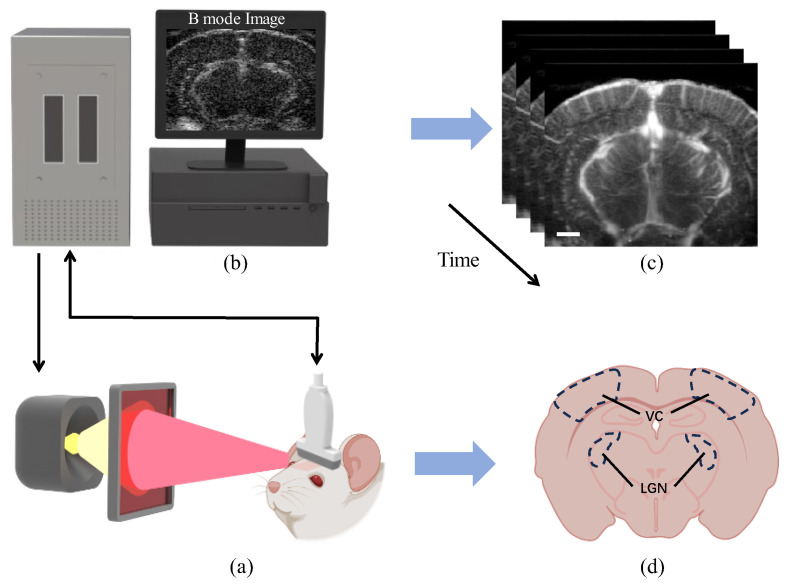
Schematic diagram of the fUS scan of rat brain. (**a**) Schematic diagram of optical stimulation and ultrasound data acquisition, (**b**) Verasonics Vantage 256 system, (**c**) Power Doppler image of rat brain, and (**d**) Visual system of rat brain. Black dashed lines delimit the VC and LGN areas. Scale bar = 1 mm.

Ultrasound imaging was performed at 15 MHz using an L22-14v linear array connected to a Verasonics system (Vantage 256, Seattle, WA, USA). To obtain a high-quality ultrasound image, we used angled ultrasound plane waves ranging from −10° to 10° at 2° intervals, with a pulse repetition frequency of 5500 Hz. This resulted in a 500 Hz frame rate after coherent compounding. We first acquired the RF signals using the Verasonics system and saved them on a high-speed SSD. Then, we used MATLAB (MATLAB Release 2022, The MathWorks, Natick, MA, USA) for offline processing to beamform the RF signals. To separate blood signal from motion artifacts in these datasets, we used an advanced Singular Value Decomposition (SVD)-based spatiotemporal clutter filter technique [25]. More specifically, the ensembles of 250 compounded frames were acquired, and the 12 first singular values were removed to filter out noise and tissue motion to produce power Doppler images of the rat brain (see Figure 2C). Ultimately, the Power Doppler intensity was derived through incoherent temporal averaging of the blood signal at each pixel, according to the following formula:

(1) $$I=\frac{1}{N}\sum_{i=1}^{N}S_{B}^{2}\left(t_{i}\right)$$

where *N* is the number of samples acquired and *S_B_* the filtered signal [26]. 

### 2.6. Ultrasound Flow Imaging Data Analysis

After the Power Doppler data were acquired, the neural activity maps were reconstructed by calculating the cross-correlation coefficient between the Power Doppler and the pre-defined stimulus pattern. The resulting neural activation maps were then generated, displaying correlation scores superimposed on the averaged grayscale Doppler images. The threshold of the correlation score was set at a coefficient r > 3σ, where σ is the spatial STD of the correlation score in the non-activated region of the brain. This method ensured that only statistically significant changes in blood flow, reflective of actual neural activity, were considered in the final analysis. Furthermore, we quantified the changes in cerebral blood volume (CBV) during the period of neural response. CBV was measured as the percentage change from the baseline volume, offering insights into the hemodynamic response associated with neural activity.

## 3. Results

### 3.1. EEG Measurements of Visual Cortex

**Figure 3:**
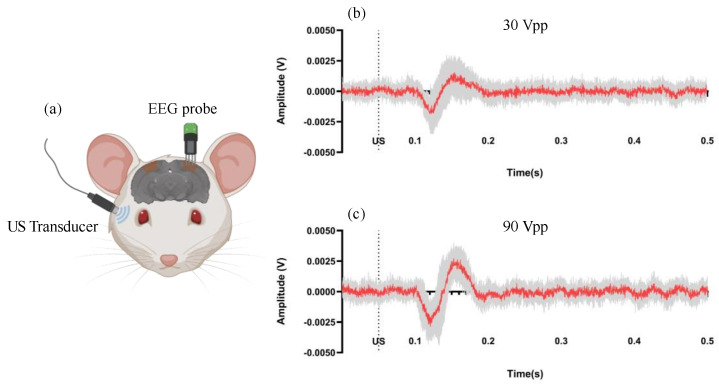
Schematic diagram (**a**) of the EEG measurement of the visual cortex with (**b**) 30 Vpp and (**c**) 90 Vpp ultrasound stimulation.

EEG results can be seen in Figure 3. The neuronal response can be evoked by ultrasound stimulation. The dashed line in the graphs indicates the onset time of the ultrasonic stimulus. The red line in each graph represents the mean response from 10 trials, while the gray area depicts the data range across these trials. Overall, the EEG response to the 90 Vpp ultrasonic stimulus exhibited a greater amplitude than the response to the 30 Vpp stimulus.

### 3.2. Response of Visual Areas of the Brain to White Light Stimulation

**Figure 4:**
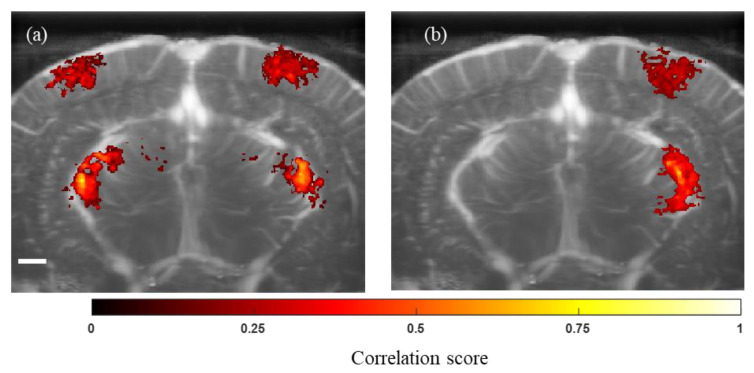
Ultrasound imaging of the higher visual centers in rat brain during white light stimulation. (**a**) Activation of both hemispheres is observed during bilateral white light stimulation. (**b**) Unilateral white light stimulation caused the activation of the contralateral in the brain’s visual center alone. Scale bar = 1 mm.

For white light stimulation, we used full-field white light flashes at a frequency of 3 Hz to stimulate the rat eyes. Each experiment included five sets of light stimulation (total period of light stimulation, approximately 30 s) followed by 60 s interlevel with dark adaptation. During bilateral light stimulation (stimulation of both eyes, Figure 4a), the rats’ LGN and visual cortex showed bilateral activation, while unilateral stimulation (stimulation of left eye alone, Figure 4b) resulted in the activation of the corresponding right hemisphere alone. These data suggest that functional ultrasound stimulation activates higher visual areas of rat brain according to the spatial location of the visual stimuli. This observation is consistent with the previously established studies on rats showing the activation of the brain’s visual areas corresponding to the contralateral visual field [27].

### 3.3. Brain Response to Different Colors and Ultrasound Stimuli in Visual Areas

In our research, we sought to delve deeper into the effects of chromatic stimulation on retinal activation in rats by modifying the white light stimulation protocol. We employed a series of optical filters, each designed to permit only a specific wavelength of light to pass through, thereby creating a spectrum of color-specific stimuli. These filters were carefully positioned 8 cm from the rats’ eyes, a distance determined to be optimal for light exposure without causing discomfort to the animal. Our experimental design was methodical and consistent across all tests similar to white light stimulation. We initiated each trial with a 60-s period of complete darkness to standardize the rats’ visual baseline. This was followed by a series of five light stimulation sessions (total period of light stimulation was 30 s for each session). After each session, a 60 s dark adaptation period was provided. This pattern was replicated for each color tested, ensuring the comparability of the data.

**Figure 5:**
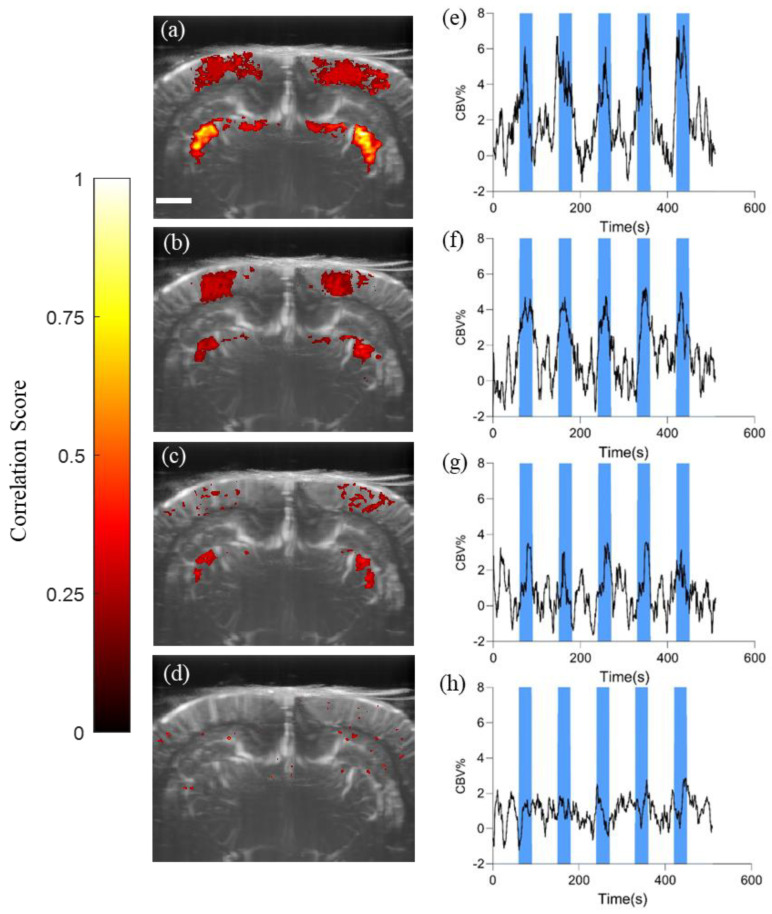
Activation maps overlaid on power Doppler images of the rat brain. (**a**) Blue light stimulation, (**b**) Yellow light stimulation, (**c**) Red light stimulation, (**d**) US stimulation; Cerebral blood volume (CBV) changes in the most responsive LGN area. (**e**) Blue light stimulation, (**f**) Yellow light stimulation, (**g**) Red light stimulation, (**h**) US stimulation. Scale bar = 2 mm; Blue block—Light-on/US on condition.

We acquired the Power Doppler signal and calculated the activation map to evaluate the correlation between light stimulation and activity in the lateral geniculate nucleus (LGN), a crucial part of the brain’s visual processing pathway. Figure 5a–c corresponds to the visual areas of neural activity that respond to blue, yellow, and redlight stimuli, respectively. The blue light stimulus resulted in the highest LGN correlation value of 0.84 ± 0.02, mean ± SEM, indicating a strong response to this wavelength. On the other hand, the yellow and red stimuli showed moderate to weak correlations, with values of 0.56 ± 0.03, mean ± SEM, and 0.37 ± 0.02, mean ± SEM, respectively (blue light vs. red light, blue light vs. yellow light, and yellow light vs. red light all showed significant differences with *p*-values < 0.001, one-way ANOVA, Tukey–Kramer multiple correction). This variation in responsiveness can be attributed to the unique photoreceptor distribution and processing pathways in the rat retina. Adding to these findings, the CBV curves on the right side of the same figure illustrate that the magnitude of CBV change was most pronounced during blue light stimulation, suggesting a heightened hemodynamic response. Conversely, the alterations observed in response to yellow and red light stimulation were comparatively modest.

Although we observed the brain’s visual regions responding to both white and colored light stimuli, the results from ultrasound stimulation shown in Figure 5d present a stark contrast: neither the visual cortex nor the LGN showed significant responses according to the activation map. We focused on the LGN region and calculated the CBV values, which also revealed changes of a lesser magnitude than those elicited by light stimulation. It is notable that during the latter three stimuli of the stimulation protocol, there were some fluctuations in CBV values concurrent with the application of ultrasound, yet these changes remained negligible.

## 4. Discussion

In this study, we utilized functional ultrasound imaging to investigate rat brain’s response to light and ultrasound stimuli. Our findings demonstrate that fUS provides high-resolution images capable of revealing cerebral blood volume (CBV) changes associated with neuronal activity in the visual cortex and lateral geniculate nucleus (LGN). In the broader context of neuroimaging research, particularly with optical methods used to study cortex blood flow, fUS displays unique advantages. When compared with Near-Infrared Spectroscopy (NIRS), though NIRS excels in temporal resolution, fUS offers significantly higher spatial resolution, which is essential for studying smaller subjects like rodents [28]. Furthermore, contrasting fUS with recent advancements in Diffuse Speckle Contrast Analysis (DSCA), which achieves deeper tissue penetration through fiber insertion, highlights fUS’s ability to provide a broader field of view and non-invasive imaging [29]. While DSCA balances spatial and temporal resolution effectively, its invasive nature and constrained field of view may limit its utility in the comprehensive monitoring of multiple brain regions. These observations underscore the versatility and superiority of fUS in neuroimaging, offering non-invasive, high-resolution imaging across diverse cerebral areas crucial for both human and animal models.

The differential response of the visual areas to various colored-light stimuli underlines the sensitivity of rat’s visual system to specific wavelengths, as evidenced by the varying correlation coefficients and CBV changes. Interestingly, the blue light elicited the strongest response, which may be reflective of the distinct spectral sensitivity of rodent photoreceptors or the differential wiring of their visual pathways when compared to other colors tested. These observations corroborate previous studies highlighting the significance of wavelength-specific retinal activation in visual perception and its underlying neural mechanism. In mouse, research indicates a non-uniform distribution of cone opsins in the retina, which suggests that color discrimination and wavelength-specific luminance contrast sensitivity may vary with location in the retina. This indicates that mice might have different sensitivities to color and light intensity [30,31,32]. Additionally, the study by Jeczmien-Lazur et al. demonstrated that rat dLGN neurons are sensitive to a wide spectrum of ultraviolet light, which aligns with our findings that the LGN region of rat brain is more sensitive to short wavelengths of light [33].

Moreover, our results indicate that while fUS is a promising modality for mapping brain activity in response to visual stimuli, the lack of significant response and CBV changes with ultrasound stimulation suggests a more complex interaction between mechanical stimuli and neuronal activity than previously understood. Qian et al. [20] demonstrated the effect of ultrasound on neural modulation by stimulating the retinas of rats with different voltages and durations of stimulation signals, detecting electrophysiological signals in the visual cortex and superior colliculus. Also, Menz et al. [34] applied focused ultrasound to the isolated retina of a tiger salamander and recorded responses in the retinal ganglion cells. These experiments collectively suggest that ultrasound can modulate neural activity. Furthermore, Lu et al.’s review article discusses [35] three primary effects of ultrasound stimulation: cavitation, acoustic radiation force (ARF), and thermal effects. They point out that ARF is the main mechanism for neural modulation; however, the precise underlying mechanism remains unclear.

Retinal cells have been reported to have several functional mechanosensitive ion channels, including TWIK-related K+ channels [36], TRP channels [37], Piezo1 [38], and calcium-selective channels [39]. TWIK-related K+ channels are sensitive to membrane stretch, acidosis, and temperature changes. TRP channels serve as temperature sensors, reacting to both increases and decreases in temperature. Piezo1 is involved in mechanotransduction, responding to physiologically relevant physical forces. Additionally, calcium-selective mechanosensitive ion channels are activated through ultrasonic stimulation, leading to calcium accumulation and amplified channel responses. Despite these channels showing potential in controlling neural activity, a specific ion channel that responds uniquely to ultrasonic stimulation has not yet been identified. Although ultrasonic stimulation can activate specific ion channels in retinal cells, the FUS imaging technique used in these experiments only shows the overall cerebral blood flow in a coronal plan. Therefore, it might not be able to detect responses from a small subset of activated cells. In contrast, using electrodes to collect signals provides more precise localization and direct measurement of cellular electrical signals within the cerebral cortex. For instance, Qian et al. [20] designed a 56-channel MEA capable of covering the SC and VC regions, offering a broader measurement scope than the FUS used in this experiment. 

Future research will aim to enhance the fUS data acquisition protocol; for example, employing a 2D array [40,41] or using a motorized stage to shift the transducer [42,43,44,45] can facilitate the collection of 3D images, thereby enhancing the imaging range and improving the fidelity of captured brain responses.

## 5. Conclusions

In conclusion, this study demonstrates the differential response of rat brain regions to visual stimuli using in vivo imaging techniques. While colored light, particularly in the blue spectrum, significantly activates the visual cortex and lateral geniculate nucleus, as evidenced by the activation map and cerebral blood volume changes, ultrasound stimulation does not produce a detectable response in these areas based on the functional ultrasound imaging data. This lack of response of the brain’s visual centers to ultrasound retinal stimulation suggests a complex interaction between mechanical stimuli and neuronal activity. Future experiments should consider refining both ultrasound stimulation and imaging techniques to enhance the detection and characterization of neural responses with ultrasound stimulation. These findings advance our understanding of the neurovascular coupling in the visual system and highlight the need for complementary imaging modalities to fully capture the range of neural responses to different stimuli.

## Data Availability

The data presented in this study are available on request from the corresponding author.
